# Clinical characteristics of coronavirus disease 2019 (COVID-19) patients in Kuwait

**DOI:** 10.1371/journal.pone.0242768

**Published:** 2020-11-20

**Authors:** Abdullah Alshukry, Hamad Ali, Yaseen Ali, Talal Al-Taweel, Mohammad Abu-Farha, Jehad AbuBaker, Sriraman Devarajan, Ali A. Dashti, Ali Bandar, Hessah Taleb, Abdullah Al Bader, Nasser Y. Aly, Ebaa Al-Ozairi, Fahd Al-Mulla, Mohammad Bu Abbas

**Affiliations:** 1 Department of Otolaryngology & Head and Neck Surgery, Jaber Al-Ahmad Hospital, Ministry of Health, South Surra, Kuwait; 2 Department of Medical Laboratory Sciences, Faculty of Allied Health Sciences, Health Sciences Center (HSC), Kuwait University, Jabriya, Kuwait; 3 Dasman Diabetes Institute (DDI), Dasman, Kuwait; 4 Gastroenterology Unit, Department of Internal Medicine, Jaber Al-Ahmad Hospital, Ministry of Health, South Surra, Kuwait; 5 Department of Anesthesia and Intensive Care, Jaber Al-Ahmad Hospital, Ministry of Health, South Surra, Kuwait; 6 Department of Pediatric Emergency, Jaber Al-Ahmad Hospital, Ministry of Health, South Surra, Kuwait; 7 Department of Preventive Health, Jaber Al-Ahmad Hospital, Ministry of Health, South Surra, Kuwait; ISI Foundation, ITALY

## Abstract

This is a retrospective single-center study of 417 consecutive patients with coronavirus disease 2019 (COVID-19) admitted to Jaber Al-Ahmad Hospital in Kuwait between February 24, 2020 and May 24, 2020. In total, 39.3% of patients were asymptomatic, 41% were symptomatic with mild/moderate symptoms, 19.7% were admitted to the intensive care unit (ICU). Most common symptoms in cohort patients were fever (34.3%) and dry cough (32.6%) while shortness in breath was reported in (75.6%) of ICU admissions. Reported complications requiring ICU admission included Sepsis (68.3%), acute respiratory distress syndrome (95.1%) and heart failure (63.4%). ICU patients were more likely to have comorbidities, in comparison to non-ICU patients, including diabetes (35.4% vs 20.3%) and hypertension (40.2% vs 26.9%). Mortality rate of cohort was 14.4% and mean age of death was 54.20 years (± 11.09) and 90% of death cases were males. Chest high-resolution computed tomography for ICU cases reveled multifocal large patchy areas of ground glass opacification mixed with dense consolidation. Cases admitted to ICU showed abnormal levels of markers associated with infection, inflammation, abnormal blood clotting, heart problems and kidney problems. Mean hospital stay for asymptomatic cases was 20.69 days ±8.57 and for mild/moderate cases was 21.4 days ±8.28. Mean stay in ICU to outcome for survivors was 11.95 days ±8.96 and for death cases 13.15 days ±10.02. In this single-center case series of 417 hospitalized COVID-19 patients in Kuwait 39.3% were asymptomatic cases, 41% showed mild/moderate symptoms and 18.7% were admitted to ICU with a mortality rate of 14.4%.

## Introduction

In early December 2019, the first clusters of coronavirus disease 2019 (COVID-19) were identified in Wuhan, China [1]. On March 11, 2020, the World Health Organization (WHO) confirmed COVID-19 as a pandemic. Since then, the disease has been spreading quickly, affecting more than 5 million people and resulting in more than 350,000 deaths, which emphasizes the threat it poses to global health [2].

Clinical manifestations of COVID-19 have shown high variability, including asymptomatic carriers, acute respiratory distress syndrome (ARDS), and pneumonia with variable severity [3–5]. Most of the identified patients experience mild symptoms, including fever, cough, dyspnea, myalgia, and fatigue. In contrast, patients with severe cases develop ARDS and severe cardiac and renal complications, which can potentially lead to death [6, 7]. Additionally, a poorer prognosis has been associated with older age, being male, and having pre-existing chronic conditions, such as hypertension, cardiovascular disease, and diabetes [8, 9]. At the same time, pediatric cases have shown a milder clinical course [10].

Countries worldwide have been affected differently by the COVID-19 pandemic, ranging from high infection and high mortality rates in countries like the USA, France, and Spain to low infection and mortality rates in countries like New Zealand [2, 11]. Multiple theories have been suggested to explain the current infection and death rate, including tighter measures, better health care systems, and genetic factors, among others [12–14]. One particular hypothesis has focused on genetic variants within the angiotensin converting enzyme 2 (ACE2) gene [15, 16]. Multiple studies have suggested that variants within the ACE2 gene can influence the viral entry into the host cell [17, 18]. In a recent study, we have shown that one variant within the ACE2 gene (N720D) was responsible for reduced infection rates in Middle Eastern countries compared with Europeans [15]. Such findings highlight the importance of investigating various populations worldwide to gain more insight into this disease.

This study is focused on presenting a cohort from Kuwait, a small country located on the northern tip of the Persian Gulf, with a population of nearly 4.5 million. Kuwait reported its first cases of COVID-19 on February 24, 2020, in passengers coming from Iran. Since then, the reported cases have been increasing exponentially, reaching over 35,000 cases at the time of this report and more than 280 registered deaths [2, 12, 19]. While the search for effective treatment and vaccine for COVID-19 continues, health care systems, including that in Kuwait, are attempting to strengthen their frontline clinical services to cope with the pandemic alongside with policymakers [13, 20].

Here, we describe the demographics, baseline comorbidities, clinical characteristics, and outcomes of a cohort of patients with COVID-19 in Kuwait. We further investigate the dynamics of certain laboratory parameters in intensive care unit (ICU) admissions in relation to clinical outcomes.

## Materials and methods

### Study design

This was a retrospective study, reviewed and approved by the standing committee for coordination of health and medical research at the Ministry of Health in Kuwait (IRB 2020/1404). The requirement of written informed consent was waived by the standing committee for coordination of health and medical research at the Ministry of Health in Kuwait due to the urgency of data collection and nature of disease being studied. All methods involving the human participants was carried out in accordance with the relevant guidelines and regulations. The medical records of all confirmed COVID-19 cases admitted to Jaber Al-Ahmad Hospital in Kuwait between February 24 and May 24, 2020 were included in the study. The COVID-19 diagnoses were established based on a positive result of real-time reverse transcriptase-polymerase chain reaction (RT-PCR) assay of nasal and/or pharyngeal swabs, in accordance with WHO interim guidance [21]. Cases were divided into four groups: asymptomatic, symptomatic with mild/moderate symptoms, ICU survivors and ICU death.

### Data collection

A total of 417 patients with confirmed COVID-19 were included in the study. All patients were hospitalized in Jaber Al-Ahmad Hospital, a major governmental hospital dedicated exclusively for COVID-19 patients. Patients’ medical records were accessed and analyzed by the research team. Epidemiological, clinical, laboratory, and radiological characteristics, in addition to treatment plans and outcomes, were accessed and obtained from the medical records.

Recorded information included demographic data, medical history including underlying comorbidities; travel history; contact tracing data; clinical chemistry; hematological laboratory findings; chest radiological images; treatments; complications; and ICU admissions, durations, and dynamics of hospital stay and outcomes. Signs, symptoms, and laboratory findings were recorded on the day of hospital admission (ward and ICU). ARDS was determined in accordance with the Berlin definition [22]. Acute kidney injury was determined in accordance with the Kidney Disease: Improving Global Outcomes definition [23]. Cardiac injury was concluded based on blood cardiac markers, electrocardiography, and/or echocardiography [7].

### Hospitalization dynamics

**Fig 1:**
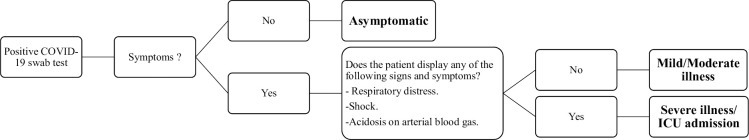
Hospitalization dynamics.

During the period in which patients were enrolled in this study, a 100% hospitalization policy was implemented at Jaber Al-Ahmad Hospital by the Ministry of Health. Any patient with a positive RT-PCR test was admitted, isolated, and put under clinical surveillance, including asymptomatic cases. Disease severity definitions were based on the following criteria; Asymptomatic cases: this group of patients included all patients with absolutely no symptoms. They have been admitted to the hospital based on a positive PCR test during a period when the rate of hospital admission was 100% for all COVID positive cases–regardless of their symptoms. These patients were all managed in the wards and required no intensive care. Symptomatic (mild to moderate); this group of patients included all patients who displayed any of the signs and symptoms described except any of the following: shock defined as SBP<90, MAP<65. (SBP = Systolic Blood Pressure / MAP = Mean Arterial Pressure), respiratory distress defined as: the presence of acute, hypoxemic respiratory failure requiring ventilatory support (respiratory deterioration leading to significantly increased work of breathing; the need for > 15L of oxygen (via non-rebreather mask; Arterial hypoxemia), presence of acidosis on Arterial Blood Gas (ABG) testing: pH < 7.30, Pa CO2 > 50 mm Hg and deterioration in the patient’s mental status. These patients were all managed in the wards and required no intensive care. Severe illness; this group included all patients that have been admitted to the ICU for ventilatory/respiratory support and/or close monitoring. The have all displayed a minimum of two of the following signs, along with the presence of radiological signs suggestive of pulmonary involvement: shock defined as SBP<90, MAP<65. (SBP = Systolic Blood Pressure / MAP = Mean Arterial Pressure), respiratory distress defined as: the presence of acute, hypoxemic respiratory failure requiring ventilatory support (respiratory deterioration leading to significantly increased work of breathing; the need for > 15L of oxygen (via non-rebreather mask; Arterial hypoxemia), presence of acidosis on Arterial Blood Gas (ABG) testing: pH < 7.30, Pa CO2 > 50 mm Hg and hemodynamic instability due to cardiogenic or septic shock and clinical/radiological/laboratory evidence of heart failure; acute cardiac injury; and acute kidney injury secondary to COVID-19 manifestation (Fig 1). Patients were discharged from the hospital when two negative PCR test results were obtained, 48 hours apart.

For ICU dynamics analysis, we divided patients into two groups: ICU survivors and ICU deaths. Clinical analysis of blood samples was performed for all patients on a daily basis. The daily values of selected laboratory parameters were averaged and plotted until an outcome was achieved.

### Statistical analysis

The variables analyzed in the study were divided into categorical and continuous variables. The categorical variables were described as frequencies and percentages, while continuous variables were presented as medians and interquartile range (IQR) values and means and standard deviations. All statistical analyses were performed using GraphPad Prism software (La Jolla, CA, USA) and SPSS (Statistical Package for Social Sciences) for Windows version 25.0 (IBM SPSS Inc., USA).

## Results

### Cohort characteristics

**Fig 2:**
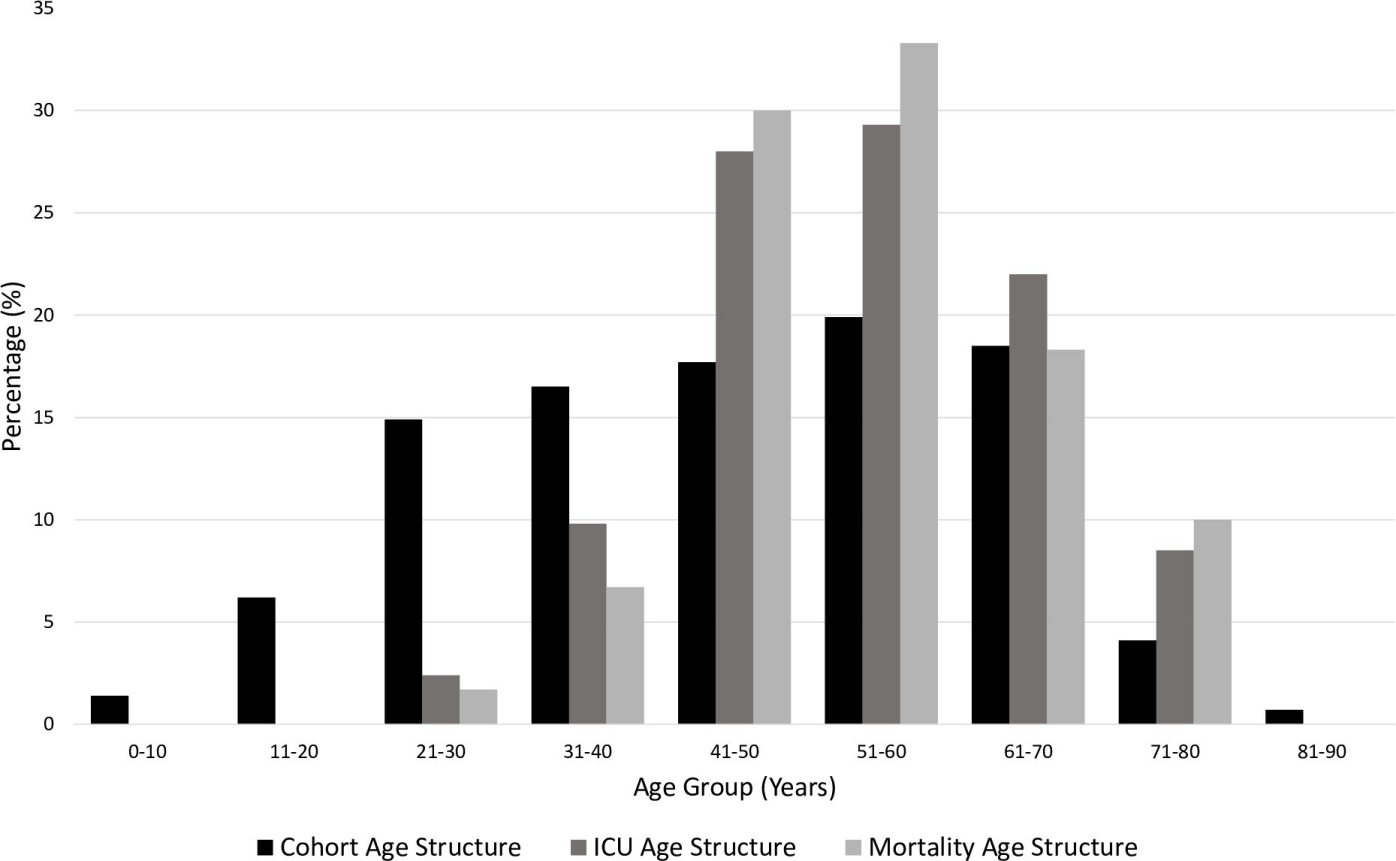
Categorical age structures. Each age group percentage is calculated by dividing the count by the total in each category. Highest ICU admissions were in the age groups (41–50 years) and (51–60 years) while highest numbers of death cases were recorded in the age group (51–60 years).

In our study, 417 hospitalized patients with COVID-19 with a positive viral RT-PCR test were enrolled. The cohort had a median age of 47 years (IQR 32–60 years), and 63% of the cohort were men. The age structure of the cohort showed that the majority of patients belonged to the 21–40 years age group, and notably, only 0.3% belonged to the 81–90 years age group (Fig 2 and Table 1). The cohort consisted of 39.3% asymptomatic patients, 41% symptomatic (mild to moderate), 5.3% admitted to the ICU who recovered, and 14.4% admitted to ICU who died (Table 2).

**Table 1:** Cohort age structure and case-fatality rate (CFR) per age group.

	No. of cases	% of total	No. of ICU admissions	% of total ICU	No. of deaths	% of total deaths	Case-fatality rate, %
All	417	100	82	100	60	100	14.4
Age groups, years							
0–10	6	1.4	0	0.0	0	0.0	0.0
11–20	26	6.2	0	0.0	0	0.0	0.0
21–30	62	14.9	2	2.4	1	1.7	1.6
31–40	69	16.5	8	9.8	4	6.7	5.8
41–50	74	17.7	23	28.0	18	30.0	24.3
51–60	83	19.9	24	29.3	20	33.3	24.1
61–70	77	18.5	18	22.0	11	18.3	14.3
71–80	17	4.1	7	8.5	6	10.0	35.3
81–90	3	0.7	0	0.0	0	0.0	0.0

**Table 2:** Cohort baseline characteristics.

	Asymptomatic	Symptomatic	ICU Survivors	ICU Death
Number N (%)	164 (39.3)	171 (41.0)	22 (5.3)	60 (14.4)
Age (Mean ± SD)	41.97 ± 19.21	44.67 ± 15.75	52.32 ± 13.51	54.20 ± 11.09
**Age Groups**				
0–10 Years	5 (3.0)	1 (0.6)	0	0
11–20 Years	20 (12.2)	6 (3.5)	0	0
21–30 Years	28 (17.1)	32 (18.7)	1 (4.5)	1 (1.7)
31–40 Years	28 (17.1)	33 (19.3)	4 (18.2)	4 (6.7)
41–50 Years	23 (14.0)	28 (16.4)	5 (22.7)	18 (30.0)
51–60 Years	24 (14.6)	35 (20.5)	4 (18.2)	20 (33.3)
61–70 Years	27 (16.5)	32 (18.7)	7 (31.8)	11 (18.3)
71–80 Years	7 (4.3)	3 (1.8)	1 (4.5)	6 (10.0)
81–90 Years	2 (1.2)	1 (0.6)	0	0
**Gender**				
Male	86 (52.4)	107 (62.6)	15 (68.2)	54 (90.0)
Female	78 (47.6)	64 (37.4)	7 (31.8)	6 (10.0)
**Signs & Symptoms**				
Fever	-	94 (55.0)	12 (54.5)	37 (61.7)
Chills	-	7 (4.1)	0 (0.0)	4 (6.7)
Dysgeusia	-	1 (0.6)	0 (0.0)	0 (0.0)
Dry Cough	-	89 (52.0)	12 (54.5)	35 (58.3)
Productive Cough	-	21 (12.4)	2 (9.1)	3 (5.0)
Headache	-	24 (14.1)	0 (0.0)	3 (5.0)
Chest Pain	-	5 (2.9)	0 (0.0)	5 (8.3)
Syncope	-	0 (0.0)	0 (0.0)	1 (1.7)
Runny Nose	-	13 (7.6)	0 (0.0)	1 (1.7)
Sore Throat	-	51 (29.8)	0 (0.0)	10 (16.7)
Shortness of breath	-	14 (8.2)	13 (59.1)	49 (81.7)
Nausea	-	6 (3.5)	3 (13.6)	3 (5.0)
Vomiting	-	4 (2.3)	2 (9.1)	4 (6.7)
Abdominal Pain	-	6 (3.5)	2 (9.1)	4 (6.7)
Diarrhea	-	9 (5.3)	2 (9.1)	2 (3.3)
Fatigue	-	20 (11.7)	4 (18.2)	16 (26.7)
Myalgia	-	37 (21.6)	2 (9.1)	9 (15.0)
**Hospital Admission**				
Days (Mean ± SD)	20.69 ± 8.57	21.40 ± 8.28	11.95 ± 8.96	13.15 ± 10.02
**Comorbidities**				
Diabetes	36 (22.2)	32 (18.8)	5 (22.7)	24 (40.0)
Hypertension	40 (24.5)	50 (29.2)	5 (22.7)	28 (46.7)
Asthma	14 (18.5)	12 (7.1)	3 (13.6)	12 (20.3)
COPD	0	0	0	1 (1.7)
Cardiovascular disease	7 (4.3)	18 (10.6)	1 (4.5)	13 (21.7)
Chronic renal disease	1 (0.6)	8 (4.7)	1 (4.5)	4 (6.7)
Malignancy	4 (2.4)	4 (2.4)	1 (4.5)	3 (5.0)

The majority (29.5%) of ICU admissions belonged to the 41–50-year age group, whereas 24.8% were 51–60 years and 24% were 61–70 years of age (Fig 2 and Table 1). No ICU admissions were recorded for the youngest age group (0–20 years).

Of 417 cases, we recorded 60 deaths, the majority of such patients belonging to the age group 51–60 years, which represented 33.3% of all deaths reported, with a case-fatality rate of 24.1% (Fig 2 and Table 1). However, the case-fatality rate was highest in those aged 71–80 years, at 35.3% (Table 1).

The patients with COVID-19 were divided into four groups. The first group was composed of asymptomatic patients. These patients had a positive viral RT-PCR but did not show any symptoms and were kept in hospital isolation until a negative viral RT-PCR was achieved, with an average hospital admission length of 20.69 ± 8.57 days (Table 2). The asymptomatic group made up 39.3% of the cohort, with an average age of 41.97 ± 19.21 years. Symptomatic cases made up 41% of the cohort. This group consisted of patients who exhibited mild/moderate symptoms but did not require ICU admission. Some 55% of these patients experienced fever, 52% presented with a dry cough, 29.8% reported a sore throat, and 21.6% described symptoms of myalgia (Table 2). Cases admitted to the ICU were divided into two groups based on outcome: the ICU survivors (composed of 22 patients, 5.3% of the cohort) and the ICU deaths (composed of 60 patients, accounting for 14.4% of the cohort) (Table 2). The pattern of symptoms in the ICU survivors’ group was similar to the symptomatic group except for the shortness of breath, which was present in 59.1% of cases (Table 2). Shortness of breath was more prevalent in the ICU deaths group (81.7%). Furthermore, this group had more comorbidities than the other groups, including diabetes, hypertension, asthma, cardiovascular disease, chronic renal disease, and malignancies (Table 2).

### Clinical findings

The clinical data analysis indicated significant differences between the COVID-19 groups. The asymptomatic group generally showed normal laboratory findings with minimal borderline abnormalities (Table 3). Numerous markers showed significant differences between the symptomatic/mild group and patients admitted to the ICU, including complete blood count (Table 3). Inflammatory markers, including procalcitonin (PCT) and C-reactive protein (CRP) showed progressive increasing patterns from the symptomatic to the ICU deaths group. Markers of blood clotting, including prothrombin time (PT) and activated partial thromboplastin time (APTT) showed prolonged timings in the group of ICU deaths.

**Table 3:** Laboratory findings of COVID-19 patients groups.

	Reference range /unit	Asymptomatic		Symptomatic		ICU Survivor		ICU Death	
		n	Median (IQR)	n	Median (IQR)	n	Median (IQR)	n	Median (IQR)
White blood cells (WBC)	3.7–10 x10^9 /L	164	6.24 (5.26–7.64)	171	6.2 (5.3–7.4)	22	8.08 (6.45–9)	60	12.68 (9.45–16.05)
Neutrophils	1.7–6 x10^9 /L	164	2.92 (2.30–3.38)	171	3.36 (2.64–4.1)	22	5.37 (4.38–6.64))	60	10.96 (8.02–14.21)
Lymphocytes	1–3 x10^9 /L	164	2.39 (1.89–2.89)	171	2.05 (1.74–2.5)	22	1.41 (1.14–1.93)	60	0.85 (0.68–1.05)
Monocytes	0.2–1 x10^9 /L	164	0.57 (0.48–0.7)	171	0.55 (0.46–0.7)	22	0.57 (0.5–0.79)	60	0.57 (0.35–0.79)
Eosinophils	0.02–0.5 x10^9 /L	164	0.15 (0.1–0.24)	171	0.12 (0.08–0.19)	22	0.13 (0.09–0.22)	60	0.05 (0.01–0.1)
Basophils	0.5% to 1%	164	0.45 (0.35–0.58)	171	0.4 (0.3–0.56)	22	0.33 (0.25–0.54)	60	0.22 (0.17–0.3)
Platelets	130–430 x10^9 /L	164	267.27 (225.04–315.52)	171	274 (224.8–331.33)	22	319.28 (269.03–388.08)	60	260.51 (204.03–316.34)
Red blood cells (RBC)	4.5–5.5 x10^12 /L	164	5.03 (4.52–5.43)	171	5.04 (4.6–5.28)	22	4.48 (4.19–4.82)	60	3.85 (3.36–4.35)
Hemoglobin (HB)	130–170 g/L	164	133.83 (120.54–145.51)	171	134.17 (123.33–145.5)	22	119.03 (105.96–128.63)	60	104.93 (87.08–120.39)
Prothrombin Time (PT)	10–13 s	49	13.4 (12.7–14.1)	44	13.35 (12.56–14.75)	17	13.63 (12.8–14.5)	54	15.23 (13.95–19.05)
Activated partial thromboplastin time (APTT)	23–30 s	49	32 (30.5–35.55)	45	32.5 (29.4–38.68)	17	32.05 (30–36.41)	54	45.2 (38.94–53.91)
D-dimer	0–500 mg/L	11	186 (155–436)	30	331.5 (212.5–483.38)	11	682 (555–1668)	40	1987 (817.25–5037.33)
C-reactive protein (CRP)	<10 mg/L	162	3.5 (2–8.69)	167	9.88 (4–38)	22	88.22 (45.52–106.62)	44	211.33 (133.83–303.03)
Procalcitonin (PCT)	<0.05 ng/ml	154	0.04 (0.03–0.06)	160	0.06 (0.03–0.09)	22	0.27 (0.08–0.46)	60	3.36 (1.13–9.61)
Urea	2.5–7.1 mmol/L	164	3.33 (2.83–4.03)	171	3.56 (2.95–4.24)	22	4.9 (4.31–7)	60	15.56 (9.56–22.97)
eGFR	>90 mL/min/1.73m^2^	147	105.8 (95.5–117.25)	165	104.43 (92.78–114.25)	22	94.54 (67.35–104.58)	60	47.63 (26.26–81.19)
Creatinine	74.3–107 mmol/L	164	61.46 (51.5–75.86)	171	70.5 (58.38–78.5)	22	78.83 (69.44–86.9)	60	193.63 (99.02–317.43)
Total Protein	60–80 g/L	164	65.33 (62.08–68.15)	171	64.67 (61.67–68.33)	22	61.6 (57.45–63.26)	60	56.36 (51.54–59.37)
Albumin	35–55 g/L	164	37.52 (35.12–40.3)	171	35.52 (32.1–38.26)	22	27.28 (26.6–30.65)	60	20.09 (18.38–23.13)
Alkaline phosphatase (ALP)	20–140 IU/L	164	62.17 (51.19–73.48)	171	57.25 (48.86–73)	22	72.51 (51.38–80.93)	60	96.4 (74.07–135.91)
Alanine aminotransferase (ALT)	7–56 IU/L	164	20.71 (15.08–28.5)	171	27.33 (19.33–42.71)	22	39.46 (31.47–62.58)	60	41.99 (29.67–84.35)
Aspartate aminotransferase (AST)	10–40 IU/L	164	21.45 (18.25–25.78)	171	24.5 (20.33–33)	22	36.4 (27.38–47)	60	56.75 (45.47–104.71)
Gamma-glutamyl transferase (GGT)	9–48 IU/L	164	15.83 (11.46–21.89)	170	23.29 (14.92–39.86)	22	45.05 (32.75–94.77)	60	76.5 (45.3–115.88)
Total Bilirubin	5–21 mmol/L	164	11.59 (9.43–14.63)	171	11.7 (9.32–14.01)	22	14.86 (12.36–17.25)	60	21.18 (14.79–33.91)
Direct Bilirubin	1.7–5.1 μmol/L	164	1.75 (1.25–2.4)	171	2.03 (1.38–2.7)	22	4.2 (3.25–5.84)	60	9.14 (5.88–18.88)
TROPONIN	0–0.4 ng/mL	5	0.01 (0.01–4)	29	2.67 (0.01–5.42)	10	5 (0.01–6.97)	25	56 (22–164.45)
Lactate dehydrogenase (LDH)	140–280 IU/L	36	172 (144.61–207.61)	48	214.43 (166.75–277)	12	325 (293.96–478.88)	27	547 (412.79–633.33)

Markers related to renal function showed significant abnormalities in the ICU deaths, including a reduced estimated glomerular filtration rate (eGFR) and increased urea (Table 3), which coincide with reported kidney injury complications in 65% of cases in this particular group (Table 4). Markers associated with heart injury, including troponin and lactate dehydrogenase, showed significant increases in patients admitted to the ICU and in particular, the ICU deaths (Table 3), which coincided with reported heart failure in 86% of cases in this specific group and cardiac injury in 44.1% of them (Table 4). D-dimer was within the normal range in the asymptomatic and symptomatic groups but was significantly elevated in the ICU group and the ICU deaths (Table 3).

**Table 4:** Complications and treatments in COVID-19 patients’ groups.

	Asymptomatic	Symptomatic	ICU Survivor	ICU Death
	Number (%)	Number (%)	Number (%)	Number (%)
	164 (39.3)	171 (41.0)	22 (5.3)	60 (14.4)
**Complications**				
Sepsis	NA	NA	10 (45.5)	46 (79.3)
ARDS	NA	NA	18 (81.8)	60 (100.0)
Heart Failure	NA	NA	3 (13.6)	49 (86.0)
Cardiac Injury	NA	NA	2 (9.1)	26 (44.1)
Kidney Injury	NA	1 (0.6)	1 (4.5)	39 (65.0)
**Treatment**				
Antibiotic	10 (6.1)	54 (31.6)	19 (95.0)	51 (94.4)
Antiviral	7 (4.2)	37 (21.6)	18 (90.0)	38 (70.4)
Antimalarial	3 (1.8)	24 (14.0)	12 (60.0)	37 (68.5)
Corticosteroids	NA	2 (1.2)	3 (15.0)	1 (1.9)
Plasma	NA	NA	NA	2 (3.8)
Extracorporeal membrane oxygenation (ECMO)	NA	NA	1 (5.0)	2 (3.7)
Intubation (Yes)	NA	NA	11 (55.0)	51 (94.4)
Tracheostomy (Yes)	NA	NA	1 (5.0)	6 (11.1)

**Fig 3:**
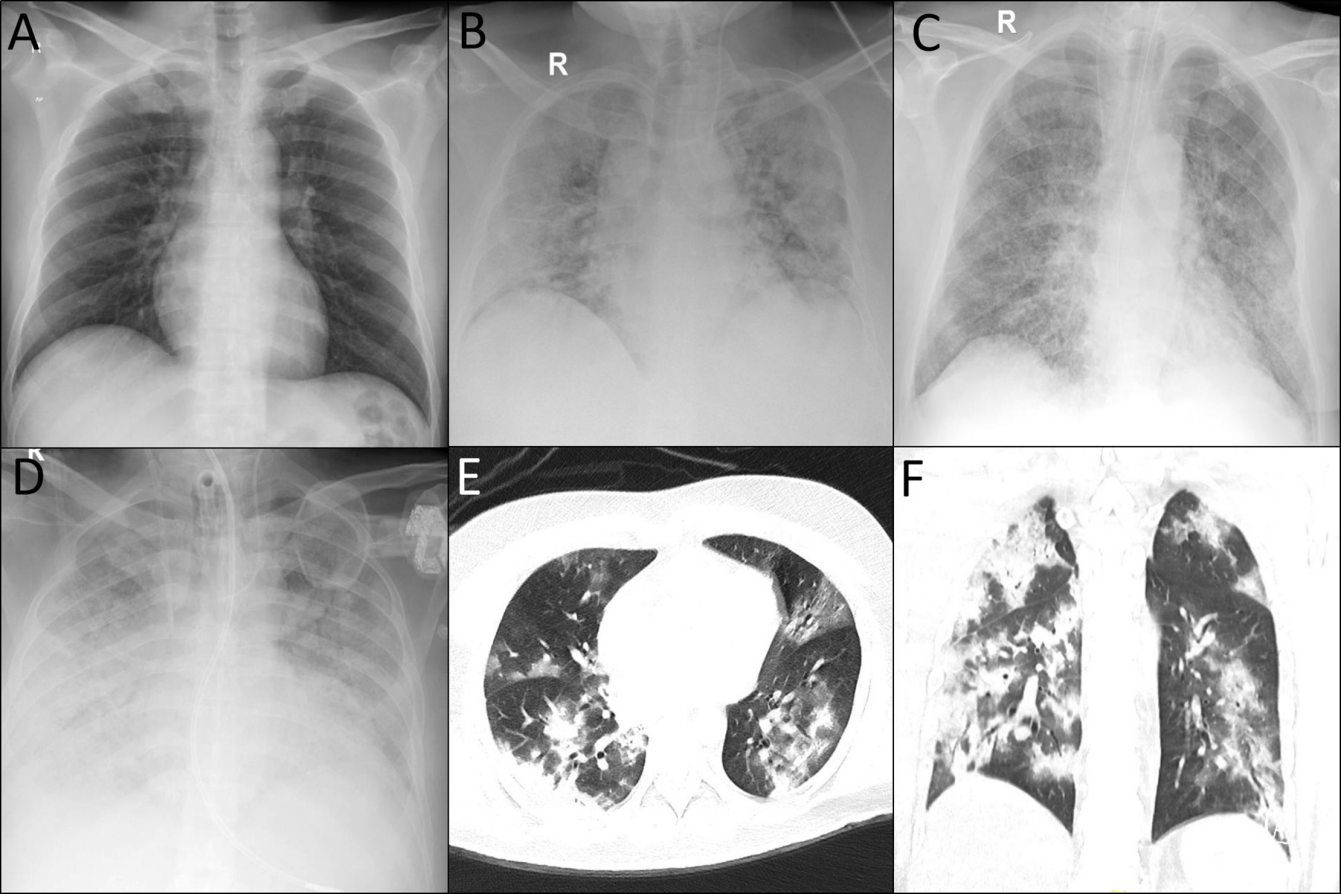
COVID-19 patients chest imaging. A. Normal chest radiography of a patient with mild to moderate COVID-19. B. Diffuse bilateral airspace opacification with patches of consolidation in an ICU survivor. C. Diffuse bilateral airspace opacification with consolidation patches in an ICU death. D. Diffuse bilateral airspace opacification with near total white out of both lung fields in an ICU death. E and F. High Resolution CT chest showing multifocal large patchy areas of ground glass opacification mixed with dense consolidations in an ICU death.

A total of 81.8% of patients in the ICU survivor group developed ARDS compared with 100% of cases in the ICU deaths (Table 3). Chest radiographs of patients in the ICU survivor and death groups showed diffuse bilateral airspace opacification with patches of consolidation (Fig 3). High-resolution chest computed tomography in the ICU deaths group showed multifocal large patchy areas of ground glass opacity mixed with dense consolidations (Fig 3). Intubation was required for 55% of the patients in ICU survivor group and in 94.4% of those in the ICU deaths group (Table 3). Treatment-wise, antibiotics such as amoxicillin, Augmentin, Rocephin, and piperacillin/tazobactam were the most commonly administered medications, with 31.6% of the symptomatic, 95% of the ICU survivors, and 94.4% of the ICU deaths group receiving them. Antiviral drugs, such as oseltamivir and lopinavir, were administered to 90% of patients in the ICU survivor group and in 70.4% of the ICU deaths group. The antimalarial drug hydroxychloroquine was administered to 60% of the ICU survivor group and 68.5% of the ICU deaths group (Table 4).

### Dynamic laboratory profiles of ICU admissions

**Fig 4:**
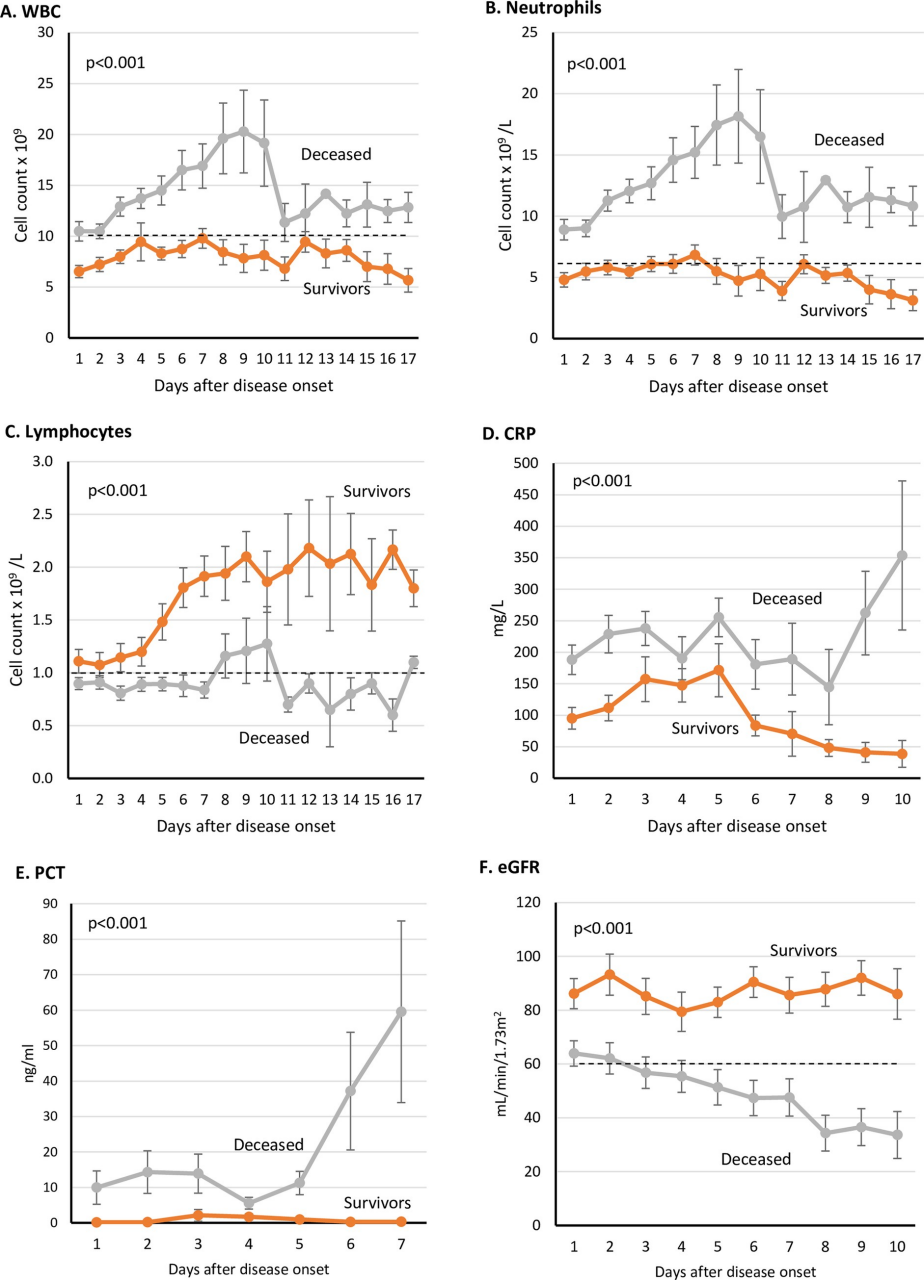
Dynamic Profile of certain laboratory markers in COVID-19 ICU patients. Laboratory tests were done on daily basis after disease onset. Dotted black lines indicate upper normal limit in A and B, and lower normal limit in C and F. (WBC: White blood cells, CRP: C-Reactive protein, PCT: Procalcitonin, eGFR: Estimated glomerular filtration rate).

Patients in the ICU deaths group showed elevated levels of white blood cells after the second day of disease onset onward. In contrast, the ICU survivor group had an average below 10 × 10^9^ cells/L (Fig 4A). A similar pattern was observed in the neutrophil count (Fig 4B). The ICU deaths group also showed lymphocytopenia compared with ICU survivors (Fig 4C). Inflammatory markers, namely CRP and PCT, showed a significant increase during ICU stay until outcome in the ICU deaths group (Fig 4D and 4E). Renal function declined progressively in the ICU deaths group from admission until outcome (Fig 4F).

## Discussion

To our knowledge, this study is one of the first detailed reports of COVID-19 clinical characteristics in Kuwait and the region. Kuwait reported its first cases from travellers arriving from Iran on February 24, 2020, followed by further cases arriving from Europe before cases from community transmission began to accumulate [12]. Patients were recruited during an early phase of the epidemic in Kuwait in which the hospitalization rate was 100% for all patients positive for SARS-CoV-2. Detecting all the cases during a specific period and from one place allows more comprehensive clinical insights, especially in relation to the inclusion of asymptomatic cases, which were isolated in the hospital with full medical surveillance.

The most prevalent symptoms in our cohort were fever, which was present in 34.3% of the cohort, dry cough (32.6%), and shortness of breath (18.2%), with the latter being more prevalent in ICU admissions (Table 2). Prevalence of symptoms varied between studies; for instance, fever was reported in 43.8% of patients on admission in a multicenter study in China [24], whereas another study conducted in the city of Wuhan found a prevalence of 98.6% [7]. These variable findings are influenced by the diagnostic criteria used and the effectiveness of surveillance strategies. It is more a question of which led to admission: a symptom or random surveillance testing based on viral PCR resulting in the detection of asymptomatic individuals.

The age structure of the cohort provided a good representation of the population in Kuwait (Fig 1 and Table 1) [25]. The asymptomatic group represented 39.3% of the reported cases, which is close to the 42.3% reported in Wuhan, the 40% reported in the USA, and the 41% reported in Italy [26, 27]. Detecting asymptomatic individuals is challenging, given these people do not raise any flags for medical attention; therefore, they can intensify infection [28]. In Kuwait, extensive viral RT-PCR testing was conducted for every passenger entering the country during the period of recruitment, in addition to active contact tracing being enacted.

The case-fatality rate (CFR) calculated for the cohort was 14.4%, which could represent an overestimation due to the proportion of ongoing cases, especially at the early stage of the outbreak [29]. In addition, undetection of asymptomatic cases and the detection bias towards more severe cases could also contributed to the overestimation of the CFR. The highest case-fatality rate was recorded in the 70–80-year age group (35.3%), which is close to what has been reported in New York City [30].

ARDS was one of the main causes for ICU admissions, given it was reported in all deaths and in 81.8% of ICU survivors. Patients admitted to the ICU were older in comparison to other groups. Our results also indicated that the ICU deaths group had a higher prevalence of comorbidities and were predominantly male (Table 2). Several studies have shown a significant association between poorer outcome and being male. This was the case in 2003 during the SARS coronavirus outbreak and now also with COVID-19 [31]. Although the reasons underlying this observation are not fully understood, the ACE2 level in males has been suggested as a possible explanation [31]. A death outcome also correlated with higher prevalence of comorbidities, as observed in the ICU deaths group, with hypertension being the most prevalent comorbidity, followed by diabetes. Similar findings have also been reported in New York [30] and Wuhan [7].

Direct comparison between groups revealed than ICU admissions and particularly the ICU death group indicated significantly lower levels of hemoglobin (Table 3). This observation has been reported previously in patients with other types of pneumonia, associated with higher mortality rate [32]. The ICU deaths group also showed prolonged PT and activated partial thromboplastin time (APPT) compared with other groups (Table 3), which indicate abnormalities in coagulation. Such abnormal coagulation parameters have been reported by previous studies, which showed an association with poor prognosis [33]. Recent recommendations advise that such prolonged APPT should not impede the use of anticoagulation therapies to prevent or treat venous thrombosis in patients with COVID-19 [34]. D-dimer levels were also significantly elevated in the ICU deaths group (Table 3). Previous studies have indicated that patients who required intubation and have higher levels of D-dimer would have a higher risk of developing pulmonary embolism [35], which highlights the need for a protocol to identify and treat such cases with anticoagulants.

For ICU admissions, a dynamic analysis of a set of markers was performed throughout the ICU stay until an outcome was reached (recovery or death). In the ICU deaths group, the WBC and neutrophil counts continued to increase until day 10, when the averages dropped but remained above the upper normal limit, which with the decreasing lymphocytes indicate active ongoing infection. PCT levels continued to increase in the ICU deaths group until an outcome was concluded, which could be caused by bacterial coinfection and has been associated with severe disease outcome [36]. CRP also followed a similar increasing pattern, which could indicate a severe inflammatory cascade possibly associated with ARDS (Table 4) and eventually leading to death [37].

Kidney injury was more prevalent in the ICU deaths group compared with the ICU survivor and symptomatic groups (65% vs. 4.5% and 0.6%, respectively). This matched the reported progressive impairment of kidney function in the ICU deaths group, as suggested by the declining eGFR low total protein and albumin and increased urea (Fig 4, Tables 3 and 4). ACE2 might play an important role in kidney involvement in COVID-19, particularly in severe cases, given it is highly expressed in the tubular epithelial cells of the kidneys [38]. When SARS-CoV-2 infects the renal epithelial cells through the ACE2 receptor, it can initiate a cascade of pathological events, including mitochondrial impairment, tubular necrosis, collapsing glomerulopathy, and eventually kidney injury and impairment [39]. Remarkably, our reported kidney injury prevalence in the ICU group exceeded what has been reported by studies in the USA and Europe, where the prevalence ranged from 20% to 40% of patients admitted to the ICU [30, 39]. This could be attributed to the high prevalence of cardiovascular disease in Kuwait, as suggested by the 41% proportional mortality [40]. Moreover, 46.7% of the patients in the ICU deaths group had a history of hypertension, which could negatively impact the deteriorating kidney function [41]. The dynamic profile of markers in ICU cases (Fig 4) could highlight possible pathological mechanisms leading to death in patients with COVID-19. Thus far, there is no effective treatment for COVID-19. Patients are provided with supportive care, and efforts are directed toward controlling the infection by antibiotics, antivirals, and antimalarial drugs without noticeable favourable outcome.

This study has a number of limitations. First, being a single-center retrospective study, it makes it susceptible to selection or referral bias. Second, no multivariable analysis was performed due to possible confounder effects. Third, reported CFR is overestimated as data was collected during the early stage of the outbreak in Kuwait.

## Conclusion

Here, we provide a detailed clinical analysis of a cohort of 417 consecutively recruited patients with COVID-19 from a single hospital in Kuwait. In total, 39.3% of patients were asymptomatic, 41% were symptomatic with mild/moderate symptoms, 5.3% were admitted to the ICU and recovered, and 14.4% died.
